# The Association between Fish Consumption and Risk of Renal Cancer: A Meta-Analysis of Observational Studies

**DOI:** 10.1371/journal.pone.0081939

**Published:** 2013-11-28

**Authors:** Hong-wei Bai, Ye-yong Qian, Bing-yi Shi, Gang Li, Yu Fan, Zhen Wang, Ming Yuan, Lu-peng Liu

**Affiliations:** Department of Urology, Institute of Organ Transplantation of PLA, 309th Hospital of PLA, Beijing, China; University of Newcastle, Australia

## Abstract

**Background:**

Several case-control studies and cohort studies have investigated the association between fish intake and renal cancer risk, however, they yielded conflicting results. To our knowledge, a comprehensive assessment of the association between fish consumption and risk of renal cancer has not been reported. Hence, we conducted a systematic literature search and meta-analysis to quantify the association between fish consumption and renal cancer.

**Methods:**

A systematic search was performed using the PubMed, Embase, and Cochrane Library Central database for case-control and cohort studies that assessed fish intake and risk of renal cancer. Two authors independently assessed eligibility and extracted data. Fixed-effect and random-effect models were used to estimate summary relative risks (RR) and the corresponding 95% confidence intervals (CIs). Subgroup analyses, sensitivity analysis and cumulative meta-analysis were also performed.

**Results:**

A total of 12 case-control studies and three cohort studies published between 1990 and 2011 were included in the meta-analysis, involving 9,324 renal cancer cases and 608,753 participants. Meta-analysis showed that fish consumption did not significantly affect the risk of renal cancer (RR=0.99, 95% CI [0.92,1.07]). In our subgroup analyses, the results were not substantially affected by study design, region, gender, and confounder adjustments. Furthermore, sensitivity analysis confirmed the stability of results.

**Conclusions:**

The present meta-analysis suggested that there was no significant association between fish consumption and risk of renal cancer. More in-depth studies are warranted to report more detailed results, including stratified results by fish type, preparation method, and gender.

## Introduction

Renal cancer accounts for almost 2% of all cancers worldwide, which consists of malignant tumors arising from the renal parenchyma and renal pelvis [1,2]. Renal cell carcinoma(RCC) accounts for about 90% of adult renal cancer and 3% of adult malignancies. The incidence of renal cancer has been steadily increasing worldwide in males and females, doubling over the past three decades[1-3]. Although cigarette smoking, obesity, and hypertension are established risk factors, the etiology of renal cancer is largely unknown[1,4]. 

Renal cancer is a multifactorial disease, with both hereditary and environmental components playing a role[5]. It has been found that diet is an important factor in the development of renal cancer[1,5]. Increased consumption of meat, especially red meat and processed meat were found to be associated with an increased risk of renal cancer[6]. As we know, fish is an important aspect of diet, and previous meta-analyses have investigated the association between fish consumption and the risk of several cancers. It was found that fish consumption could reduce the risk of colorectal cancer (OR=0.88; 95% CI, 0.80-0.95). However, there was no significant association between fish consumption and the risk of other cancers, such as pancreatic cancer, bladder cancer, prostate cancer, or esophageal cancer[7-12]. There are several case-control and cohort studies investigating the association between fish intake and renal cancer risk, however, they yielded conflicting results.. To our knowledge, there has not been any quantitative attempt to summarize the results on the possible fish–renal cancer risk association. Thus, we conducted a quantitative meta-analysis of currently available epidemiologic studies to verify this putative association. 

## Methods

### Study identification

This meta-analysis was conducted following the Preferred Reporting Items for Systematic reviews and Meta-Analyses guidelines(PRISMA)[13], as well as the meta-analysis of observational studies in epidemiology (MOOSE) guidelines[14]. A literature search was carried out using Pubmed (www.ncbi.nlm.nih.gov/sites/entrez) (1966 to May 2013), Embase (www.embase.com)(1947 to May 2013), and Cochrane Library Central database(http://onlinelibrary.wiley.com/cochranelibrary/search/)(1967 to May 2013). There was no restriction of origin and language. Search terms included: “fish” or “seafood” and ‘‘cancer(s)’’ or ‘‘neoplasm(s)’’ or ‘‘malignancy(ies)’’ and “renal” or “kidney”. Furthermore, the reference lists of each comparative study included in this meta-analysis and previous reviews were manually examined to identify additional relevant studies.

### Study selection

Two of the authors independently selected eligible case-control and cohort studies investigating the association between fish intake and renal cancer risk. Disagreement between the two reviewers was settled by discussing with the third reviewer. Inclusion criteria were: (i) used a case-control or cohort study design; (ii) evaluated the association between fish intake and renal cancer risk; (iii) presented odds ratio (OR), relative risk (RR), or hazard ratio (HR) estimates with its 95% confidence interval (CI). When there were multiple publications from the same population, only data from the most recent report was included in the meta-analysis and the remaining were excluded. Studies reporting different measures of RR like risk ratio, rate ratio, hazard ratio, and odds ratio were included in the meta-analysis. In practice, these measures of effect yield a similar estimate of RR, since the absolute risk of renal cancer is low.

### Data extraction

Two of the authors independently extracted the relevant data from each included study by using a unified data form. The items included in the data form were as follows: name of first author, publishing time, country of the population studied, study design, study period, number of cancer cases and subjects, dietary assessment method, type of fish, quantity of intake, the study-specific adjusted ORs, RRs, or HRs with their 95% CIs for the highest category of fish consumption versus the lowest, confounding factors for matching or adjustments. The 2 lists from the authors were compared, and disagreements were resolved by consensus.

### Methodological quality assessment

To assess the study quality, a 10-star system on the basis of the Newcastle-Ottawa Scale was used in which a study was judged on 3 broad perspectives as follows: selection (four items, one star each), comparability (one item, up to two stars), and exposure/outcome (three items, one star each). A ‘‘star’’ presents a ‘‘high-quality’’ choice of individual study. With consideration that there is a correlation between caloric intake and nutrient consumption, and possibly a direct or indirect causal relation between caloric intake and renal cancer risk, the scoring system was modified by adding an item in which a study with data analysis that used an energy-adjusted residual or nutrient-density model received an additional star [15]. Hence, the full score was 10 stars, and the high-quality study was defined as a study with≥7 awarded stars.

### Data synthesis and analysis

Heterogeneity was assessed using the Cochran Q and *I* ^2^ statistics. For the Q statistic, a P value<0.10 was considered statistically significant for heterogeneity; for the *I* ^*2*^ statistic, heterogeneity was interpreted as absent (*I* ^2^: 0%–25%), low (*I* ^2^: 25.1%–50%), moderate (*I* ^2^: 50.1%–75%), or high (*I* ^2^: 75.1%–100%)[16]. To better investigate the possible sources of between-study heterogeneity, a meta-regression analysis was performed[17]. Some studies presented individual risk estimates according to the different types of fish, and did not report the effect of total fish consumption. In this situation, the study-specific effect size in overall analysis was calculated by pooling the risk estimates of the various fish types, using the inverse-variance method[18]. For studies that reported results separately for males and females, but not combined, we pooled the results using a fixed-effect model to obtain an overall combined estimate before combining with the rest of the studies[19]. Subgroup analyses were carried out according to (i) study design ( cohort study versus population based case-control study versus hospital based case-control study), (ii)geographic location (Europe versus North America versus others), (iii) gender (male versus female), (iiii) number of adjustment factors (n ≥ 7 versus n ≤ 6), adjustment for alcohol intake (yes versus no), adjustment for total energy intake (yes versus no). Pooled RR estimates and their corresponding 95 % CIs were calculated using the inverse variance method. When substantial heterogeneity was detected(I^2^≥50%), the summary estimate based on the random-effect model (DerSimonian-Laird method)[20] was reported, which assumed that the studies included in the meta-analysis had varying effect sizes. Otherwise, the summary estimate based on the fixed-effect model (the inverse variance method)[21] was reported, which assumed that the studies included in the meta-analysis had the same effect size. We carried out sensitivity analysis by excluding one study at a time to explore whether the results were significantly influenced by a specific study. Cumulative meta-analysis was also performed to identify the change in trend of reporting risk over time. In cumulative meta-analysis, studies were chronologically ordered by publication year, then the pooled RRs were obtained at the end of each year. Publication bias was assessed using Begg and Mazumdar adjusted rank correlation test and the Egger regression asymmetry test[22,23]. All analyses were performed using Stata version 11.0 (StataCorp, College Station, TX). 

## Results

### Literature search and study characteristics

**Figure 1:**
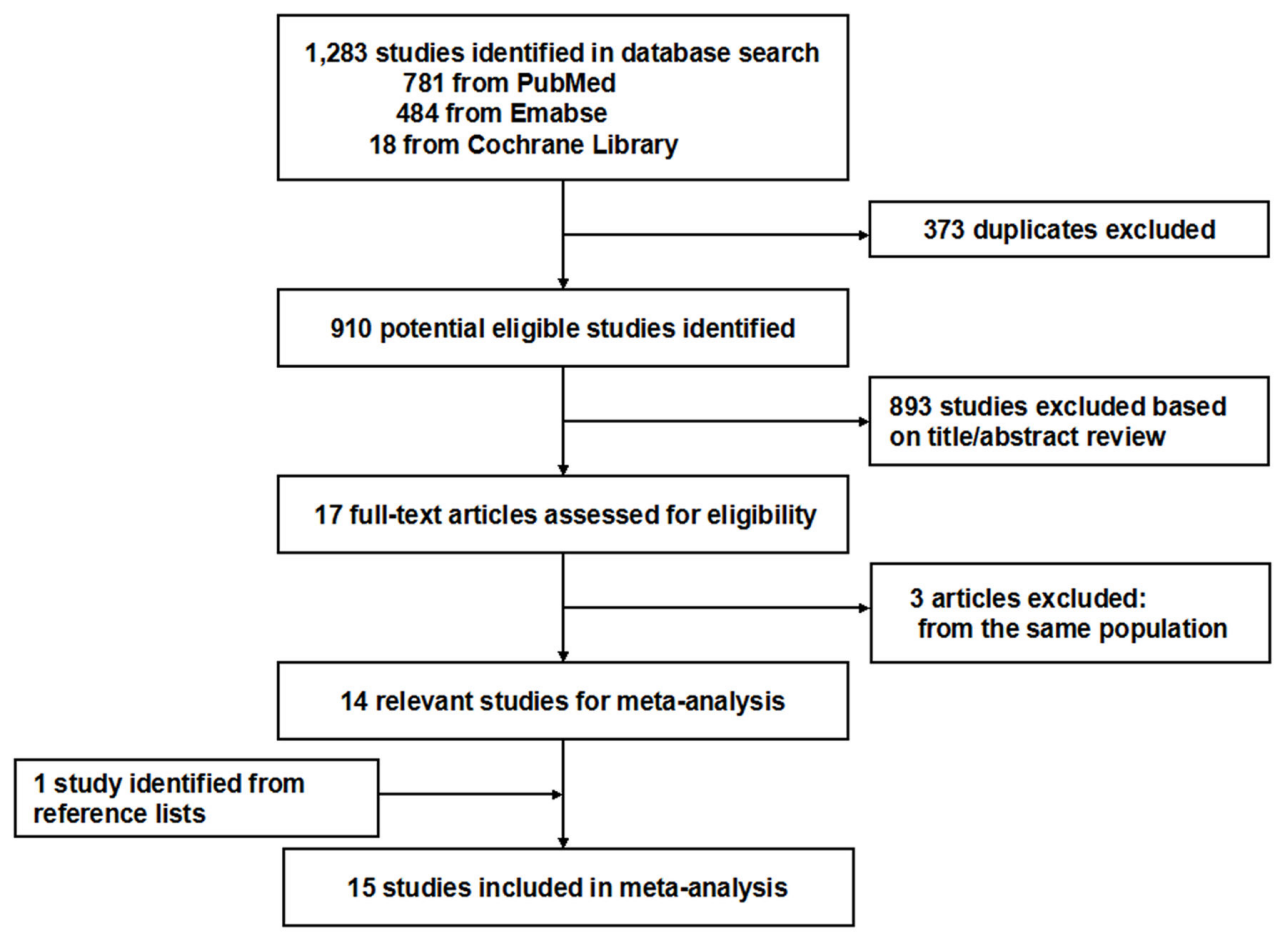
Flow diagram of screened, excluded, and analyzed publications.

A flow diagram that shows how we located relevant studies is presented in Figure 1. A total of 1,283 citations were identified from the three databases. On the basis of the title and abstract, we identified 17 papers. After reviewing the full text, three studies were excluded, because they were from the same population[24-26]. One study was identified from reference lists[27]. At last, the remaining 15 studies published between 1990 and 2011 were included in the meta-analysis, involving a total of 608,753 participants and 9,324 renal cancer cases. Of these 15 studies, seven were population-based case-control studies[28-34], five were hospital-based case-control studies[35-39], and the remaining three were cohort studies[27,40,41]. Four studies were conducted in North America[27,28,33,34], nine in Europe [29,30,32,35-37,39-41], one in Asia[38], and the remaining one study was a multi-center study which was conducted in Australia, Denmark, Sweden and the United States[31]. Almost all studies adjusted for smoking status and body mass index(BMI), and about half of the included studies adjusted for alcohol drinking status (Baseline data and other details are shown in Table 1). Table S1 summarizes the quality scores of cohort studies and case-control studies. The Newcastle-Ottawa Scale scores for the included studies ranged from 6 to 10, with a median 7. The median scores of cohort studies and case-control studies were 8 and 7, respectively. 11 studies were deemed to be of a high quality (≥7).

**Table 1:** Characteristics of studies included in the meta-analysis.

Author	Publication year	Country	Study design	Study period	Methods used for dietary assessment	Cases/Subjects	Type of fish	Units and comparison groups	Confounders for adjustment
Daniel CR	2011	USA	cohort study	1995–1996	FFQ 124 items	2,065/492,186	Total fish	Q5 vs Q1	meat intake, age, sex, education, marital status, family history of cancer, race, BMI, smoking status, frequency of vigorous physical activity, menopausal hormone therapy in women,intake of alcohol, fruit, vegetables, and total energy
Wilson RT	2009	Finland	cohort study	1985-2002	FFQ 203 items	228/27,111	Total fish, salted/canned fish	g/day≤21.0 vs >50.7	hypertension, smoking, and BMI, education and place of residence
Hu J	2008	Canada	population based case-control study	1994-1997	FFQ 69 items	1,345/6,384	Total fish, smoked fish	4Q vs 1Q	age, province, education, BMI, sex, alcohol use, smoking, total of vegetable and fruit intake, and total energy intake
Hsu CC	2007	Eastern and Central Europe	hospital based case-control study	1999-2003	FFQ 23 items	1,065/2,574	Total fish	Tertile 3 vs Tertile 1	age, country, gender, tobacco smoking, education, BMI, hypertension medication use, alcohol consumption, and vegetable consumption
Bravi F	2007	Italy	hospital based case-control study	1992-2004	FFQ 40 items	767/2,301	Total fish	3Q vs 1Q	sex, age, period of interview, education, tobacco smoking, alcohol drinking, BMI, family history of kidney cancer, and total energy intake.
Wolk A	2006	Sweden	cohort study	1987-1990	FFQ 67 items	150/61,433	Total fish, fatty fish, and lean fish	Servings/week≥1 vs 0	education, BMI, intakes of total energy, alcohol, total meat, fruits, and vegetables, fatty fish and lean fish were mutually adjusted
Fernandez E	1999	Italy	hospital based case-control study	1983-1996	FFQ 37 items	190/8,180	Total fish	Servings/week≥2 vs <1	age, sex, area of residence, education, smoking, alcohol consumption, and BMI
Lindblad P	1997	Sweden	population based case-control study	1989-1991	FFQ 63 items	379/729	Total fish	3Q vs 1Q	age, sex, BMI, cigarette smoking, and educational level
Boeing H	1997	Germany	population based case-control study	1989-1991	FFQ 122 items	277/563	Total fish	high vs low	age, gender, educational status, tobacco smoking and alcohol consumption
Wolk A	1996	Australia, Denmark, Sweden and the United States	population based case-control study	1989-1991	FFQ 63-205 items	1,185/2,711	Total fish	4Q vs 1Q	age, sex, study center, BMI and smoking
Mellemgaard A	1996	Denmark	population based case-control study	1960-1970	FFQ 92 items	351/691	Total fish	Servings/week≥1 vs 0	age, smoking, BMI and socio-economic status
Kreiger N	1993	Canada	population based case-control study	1986-1987	self-administered questionnaire	518/1,899	Total fish	high vs low	age, active cigarette smoking status, and combined Quetelet index
McLaughlin JK	1992	China	population based case-control study	1987-1989	FFQ 65 items	154/311	Total fish	high vs low	age, education, cigarette smoking, and BMI
Talamini R	1990	Italy	hospital based case-control study	1986-1989	FFQ 14 items	240/665	Total fish	high vs low	age, sex, education, area of residence, and BMI
Maclure M	1990	USA	population based case-control study	1976-1983	mail questionnaire	410/1,015	Total fish	high vs low	age, sex, education, income, religious background, quetelet index, hypertention, heart disease, kidney stone, kidney infection

BMI: body mass index; FFQ = food frequency questionnaire

### Main analysis

**Figure 2:**
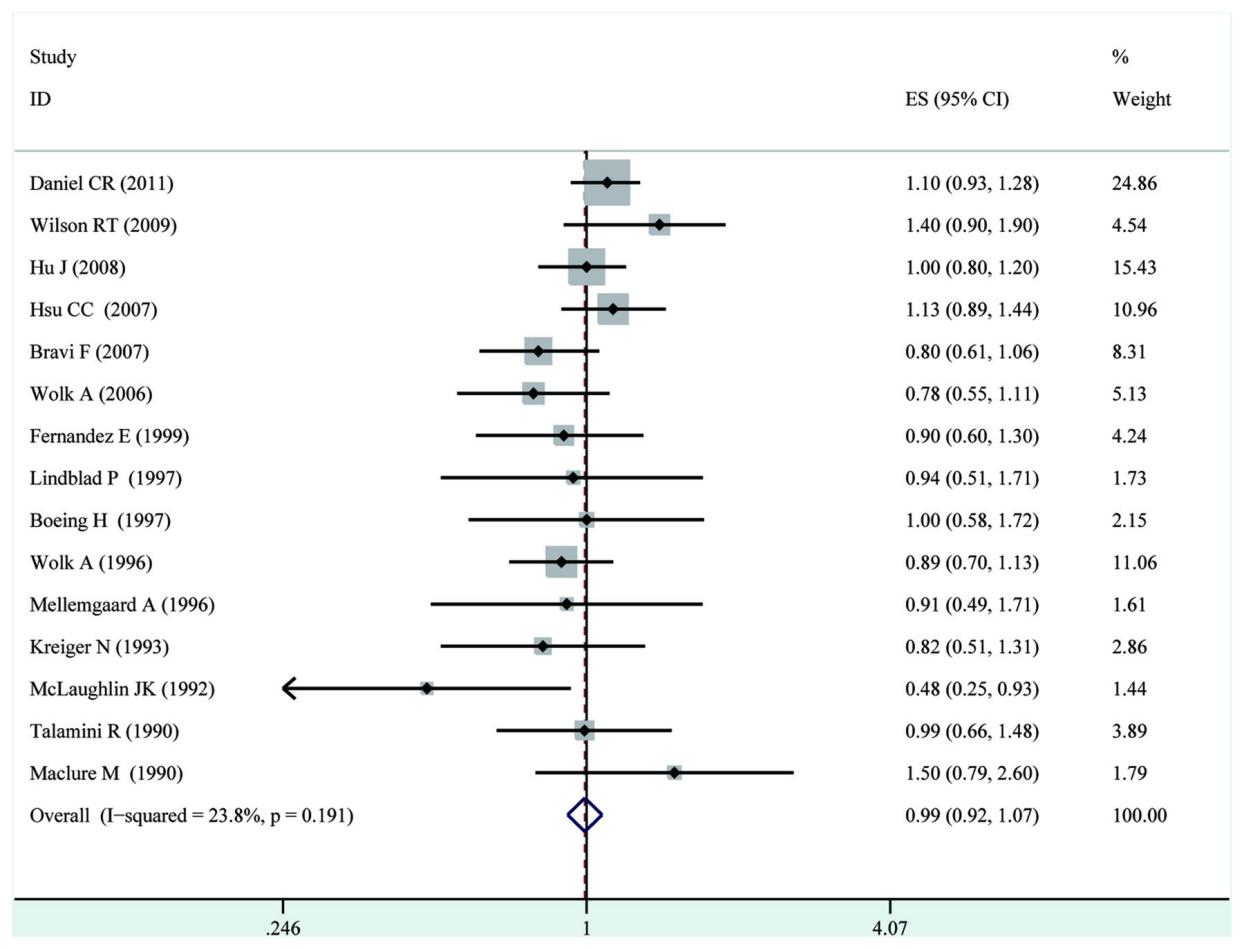
Forest plot: overall meta-analysis of fish consumption and renal cancer risk. Squares indicated study-specific risk estimates (size of square reflects the study-statistical weight, i.e. inverse of variance); horizontal lines indicate 95% confidence intervals; diamond indicates summary relative risk estimate with its corresponding 95% confidence interval.

Because of statistically significant heterogeneity was not observed (*I* ^2^ =23.8%, p = 0.19), a fixed-effects model was chosen over a random-effects model, and we found that fish consumption did not significantly affect renal cancer risk(RR=0.99, 95% CI [0.92,1.07]). Both multivariable adjusted RR estimates with 95 % CIs of each study and combined RR are shown in Figure 2. 

### Subgroup analyses, sensitivity analysis, cumulative meta-analysis, and meta-regression analysis

No statistically significant association was detected between fish consumption and renal cancer risk among cohort studies(RR=1.03, 95% CI [0.80, 1.33]), population based case-control studies (RR=0.94, 95% CI [0.82, 1.07]), or hospital based case–control studies (RR=0.96, 95% CI [0.83, 1.12]), presented in Table 2.

**Table 2:** Meta-analysis of fish consumption and renal cancer risk.

	No. of studies	Pooled estimate		Tests of heterogeneity	
		RR	95% CI	P value	I^2^(%)
All studies	15	0.99	0.92-1.07	0.19	23.80
Study design					
Cohort	3	1.03	0.80-1.33	0.03	79.80
Population based case–control	8	0.94	0.82-1.07	0.40	4.10
Hospital based case–control	4	0.96	0.83-1.12	0.31	15.80
Geographic location					
Europe	9	0.98	0.86-1.10	0.36	8.70
North America	4	1.01	0.84-1.20	0.29	19.40
Other	2	0.83	0.66-1.04	0.09	66.40
Gender					
Male	3	0.72	0.47-1.10	0.10	56.30
Female	4	0.78	0.58-1.03	0.73	0.00
Adjusted for confounders					
Number of adjustment factors					
n ≥ 7 confounders	7	0.97	0.86-1.09	0.21	30.00
n ≤ 6 confounders	8	0.94	0.81-1.09	0.25	22.30
Major confounders adjusted					
Alcohol					
yes	7	0.96	0.85-1.07	0.41	0.30
no	8	0.97	0.83-1.12	0.13	37.40
Total energy intake					
yes	4	0.90	0.77-1.04	0.31	14.80
no	11	1.00	0.89-1.12	0.24	21.00
Processed fish	2	0.91	0.70-1.19	0.25	25.30

RR= relative risks; CI=confidence intervals

**Figure 3:**
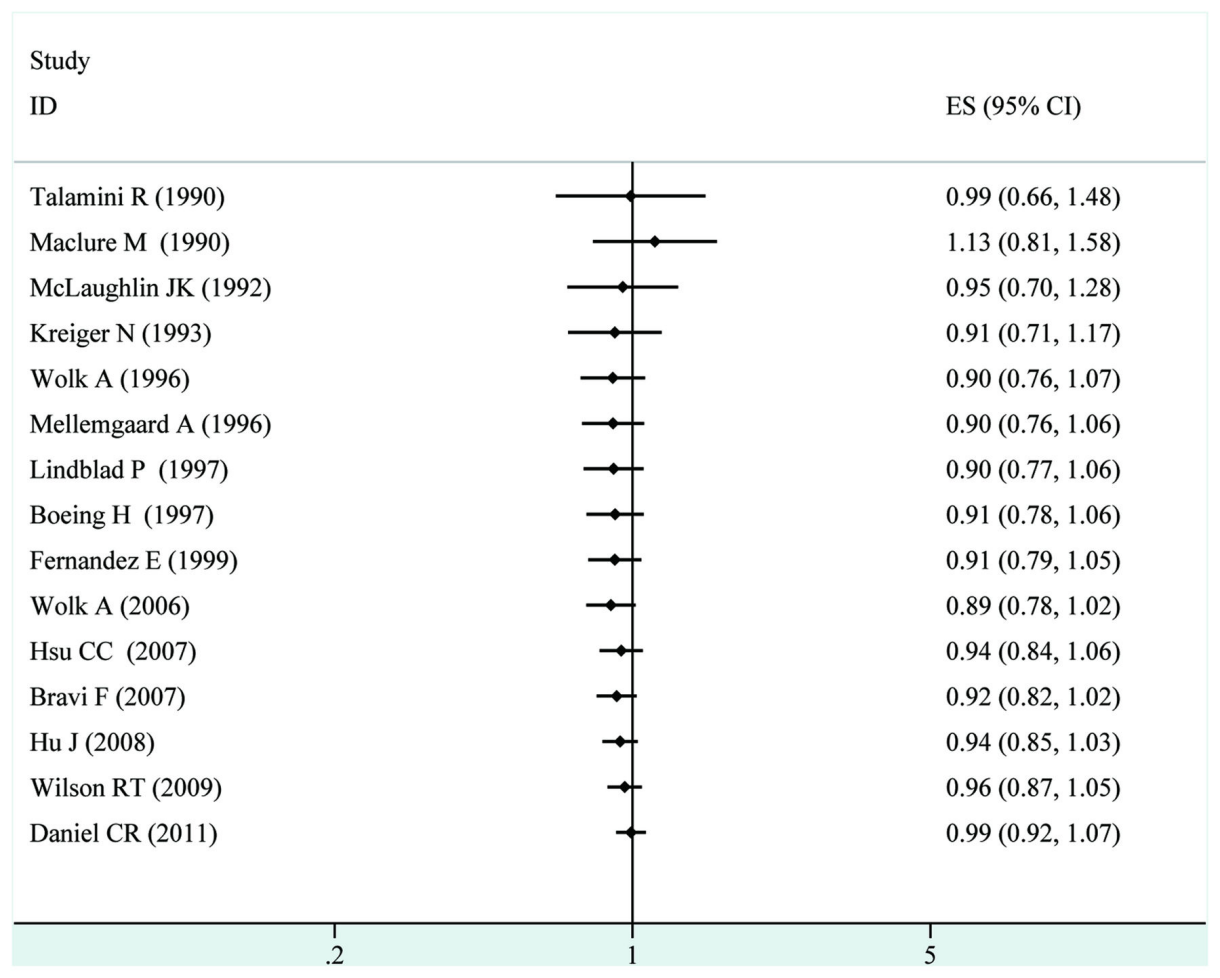
Forest plot: cumulative meta-analysis of fish consumption and renal cancer risk.

When stratified the various studies by study population, we found no significant association among studies conducted in Europe (RR= 0.98, 95%CI [0.86, 1.10]), North America (RR= 1.01, 95%CI [0.84, 1.20]), or other countries (RR= 0.83, 95%CI [0.66, 1.04]). No significant association was observed in both male(RR= 0.72, 95%CI [0.47, 1.10]) or female population(RR= 0.78, 95%CI [0.58, 1.03]). When we examined whether the associations differed by adjustment for alcohol intake, or total energy intake status, the associations did not vary by these factors. Further, it was observed that studies with higher control for potential confounders ( n ≥ 7) as well as studies with lower control (n ≤ 6) presented no significant association between fish intake and renal cancer risk (RR=0.97, 95% CI[0.86, 1.09] and RR=0.94, 95% CI[0.81, 1.09], respectively)(shown in Table 2). To test the robustness of association and characterize possible sources of statistical heterogeneity, sensitivity analysis were carried out by excluding studies one-by-one and analyzing the homogeneity and effect size for all of rest studies. Sensitivity analysis indicated that no significant variation in combined RR by excluding any of the study, confirming the stability of present results. A cumulative meta-analysis of total 14 studies was carried out to evaluate the cumulative effect estimate over time. In 1990, Talamini R and Maclure M et al reported an effect estimate of 1.13 (95% CI [0.81, 1.58]). Between 1991 and 1999, seven studies were published, with a cumulative RR being 0.91(95% CI [0.79, 1.05]). Between 1999 and 2009, five more publications were added cumulatively, resulting in an overall effect estimate of 0.99 (95% CI [0.92, 1.07])(Figure 3). To better investigate the possible sources of between-study heterogeneity, a meta-regression analysis was performed. Study design, geographic area, control source, publication year, control for confounding factors, which may be potential sources of heterogeneity, were tested by a meta-regression method. However, meta-regression revealed that none of the above factors were responsible for the between-study heterogeneity.

### Publication bias

**Figure 4:**
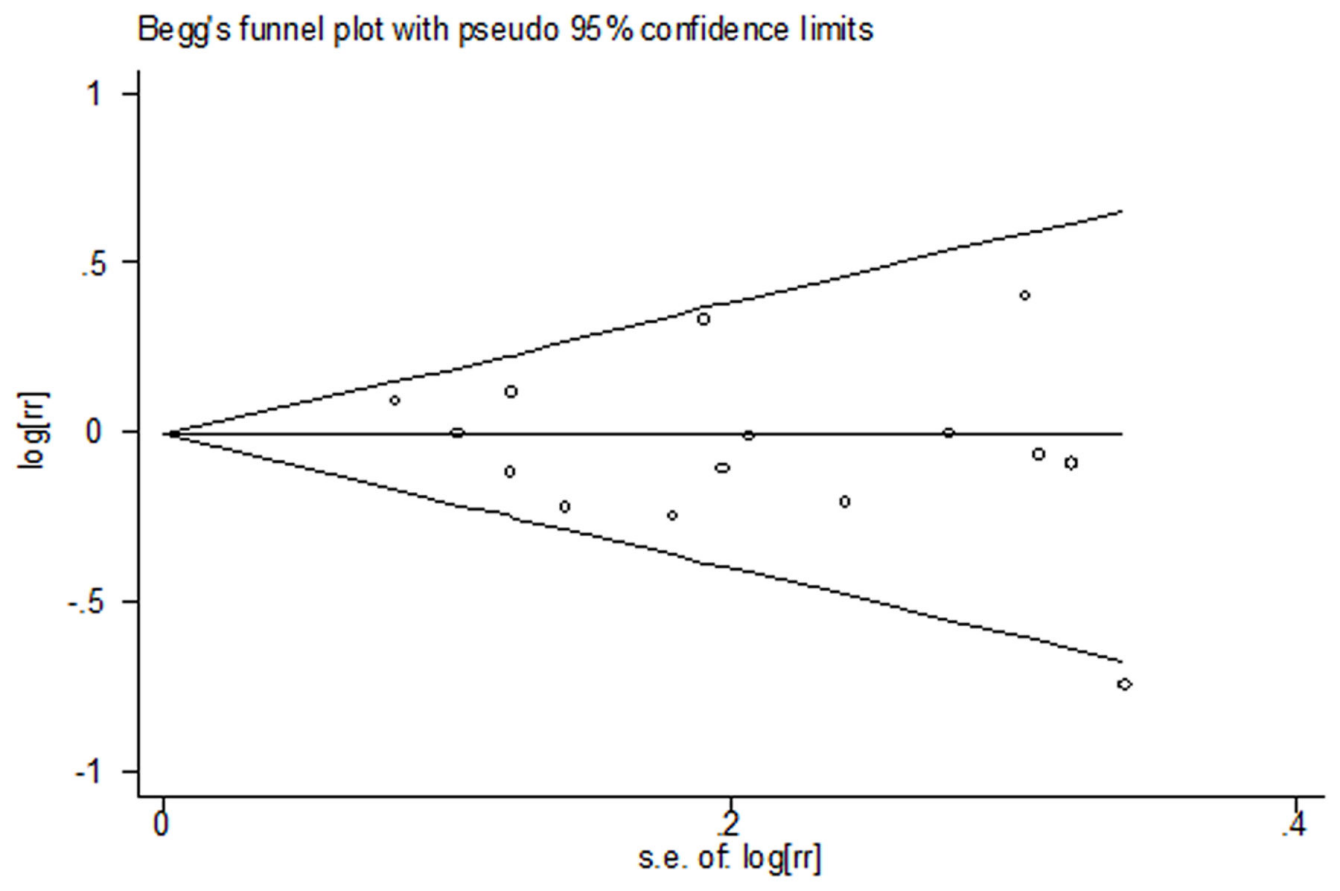
Funnel plot for publication bias in the studies investigating risk for renal cancer associated with fish intake.

In the present meta-analysis, no publication bias was observed among studies using Begg’s P value (P = 0.40); Egger’s (P = 0.38) test, which suggested there was no evidence of publication bias (Figure 4).

## Discussion

The present meta-analysis included 15 observational studies currently available (12 case–control studies and three cohort studies), involving a total of 608,753 participants and 9,324 renal cancer cases. There was no statistically significant heterogeneity among the 15 studies, so a fixed-effects model was chosen over a random-effects model. Finally, we found that fish consumption did not significantly affect the risk of renal cancer(comparing the highest with the lowest category). In our subgroup analyses, the results were not substantially affected by study design, geographic location, gender, or confounder adjustments. Cohort and case–control studies alone showed no significant association between fish consumption and the risk of renal cancer. However, we should notice that there were only three cohort studies investigating the association between fish intake and renal cancer risk. That number was rather low to draw firm conclusions. Furthermore, most of the included studies didn’t reported results separately for males and females. So, future studies should reported results separately for males and females. Sensitivity analysis indicated that an omission of any studies did not alter the magnitude of observed effect, suggesting a stability of our findings. Cumulative meta- analysis showed that the estimates gradually became consistent, and the corresponding CIs narrowed down with the increase of the number of included studies in the order of publication year. Moreover, the results of Begg’s test and Egger’s test did not support the existence of significant publication bias. 

Fish consumption has both anticarcinoma and carcinogenic effects. As we know, fish oil is rich source of n-3 fatty acids. Previous animal model studies have shown that n-3 polyunsaturated fatty acids were linked to the reduction of the progression of cancer cells [42,43]. Multiple mechanisms are involved in this chemopreventive activity, including suppression of neoplastic transformation, cell growth inhibition and enhanced apoptosis, and antiangiogenicity [44-46]. On the other hand, fish consumption is positively correlated with blood levels of dioxin, polychlorinated biphenyls, cadmium, mercury, and lead[47-49]. Cadmium, mercury, and lead are known nephrotoxicants which will induce oxidative stress and damage to the proximal renal tubule, the location where nearly renal cancer arises [50,51]. Previous studies have shown that cadmium, mercury, and lead were associated with an increased risk of renal cancer[50,52]. Maybe the combination of anticarcinoma and carcinogenic effect leads to the nonsignificant association between fish consumption and renal caner risk found in our meta-analysis.

Although we haven’t found significant association between processed fish intake and increased renal cancer risk, we should notice that there were only two studies investigating processed fish and renal cancer risk, that number was rather low to draw firm conclusion. As we know, processed fish is rich in chemical carcinogens, such as nitrites, heterocyclic amines, 2-chloro-4-methylthiobutanoic acid, and so on, which may be associated with an increased risk of renal cancer. So more studies are needed to confirm the association between processed fish consumption and the risk of renal cancer in the future.

A study of women in Sweden by Wolk et al. [41]reported a reduced risk of renal cancer with higher fatty fish (salmon, herring, sardines, and mackerel) consumption. The possible reason is that there are large differences between fatty fish and lean fish in the content of omega-3 fatty acids and vitamin D. Lower serum vitamin D levels have been found to be associated with development and progression of renal cancer[53]. This was the only study investigating the association between fatty fish and the risk for development of renal cancer, so the association is needed to be confirmed by more studies in the future, especially in male population. 

The strength of the present meta-analysis lies in a large sample size (608,753 participants and 9,324 renal cancer cases) and no significant evidence of publication bias. Two investigators independently performed the article identification, data extraction, and verification and resolved all discrepancies. Most studies adjusted for some important potential confounders, including age, sex, smoking status, and BMI. Furthermore, our findings were stable and robust in sensitivity analysis. However, several limitations to this meta-analysis should be noted. Firstly, as a meta-analysis of observational data, the possibility of recall and selection biases can’t be ruled out. Compared with case-control studies, cohort studies are less susceptible to bias due to their nature. However, the present meta-analysis included only three cohort studies, so more prospective cohort studies are need to confirm the association in the future. Secondly, we haven’t searched for unpublished studies, so only published studies were included in our meta-analysis. Therefore, publication bias may have occurred although no publication bias was indicated from both visualization of the funnel plot and Egger’s test. Thirdly, most of the included studies haven’t adjusted for hypertension, red and processed meat consumption, which are associated with an increased risk of renal cancer[6,54]. Lastly, different types of fish(lean fish and fatty fish, fresh fish and processed fish) may have different effects on renal cancer, however, we can’t do detailed subgroup meta-analysis for a lack of data. Although we assessed processed fish and renal cancer risk, the number of included studies was rather low to draw firm conclusion. Further, different processing methods may influence the effect on renal cancer. 

In conclusion, the present meta-analysis suggested that there was no significant association between fish consumption and renal cancer risk. More in-depth studies are warranted to report more detailed results, including stratified results by fish type, preparation method, and gender.

## Supporting Information

Table S1 **Methodologic quality of observational studies included in the meta-analysis.**
(DOC)

Checklist S1 **PRISMA checklist of this meta-analysis.**
(DOC)
