# Supplementary material for: The Association between Fish Consumption and Risk of Renal Cancer: A Meta-Analysis of Observational Studies
Source: PLoS One. 2013 Nov 28;8(11):e81939. doi: 10.1371/journal.pone.0081939 (PMC3842978; doi:10.1371/journal.pone.0081939)
Supplement: Table S1 — Methodologic quality of observational studies included in the meta-analysis. (DOC) [file pone.0081939.s001.doc]

**Methodologic quality of case-control studies included in the meta-analysis**

| Study and year | Selection | | | | Comparability | | Exposure | | | Data analysis that used an energy-adjusted residual or nutrient-density model | Total quality scores |
| --- | --- | --- | --- | --- | --- | --- | --- | --- | --- | --- | --- |
|  | Adequate definition of cases | Representativeness of cases | Selection of control subjects | Definition of control subjects | Study controls for age / gender | Study controls for additional factors | Exposure assessment | Same method of ascertainment for cases and controls | Non-Response rate |  |  |
| Hu J 2008 | ☆ | ☆ | ☆ | － | ☆ | ☆ | ☆ | ☆ | ☆ | ☆ | 9 |
| Hsu CC 2007 | ☆ | － | － | ☆ | ☆ | ☆ | － | ☆ | ☆ | － | 6 |
| Bravi F 2007 | ☆ | － | － | ☆ | ☆ | ☆ | ☆ | ☆ | ☆ | ☆ | 8 |
| Fernandez E 1999 | ☆ | ☆ | － | ☆ | ☆ | ☆ | ☆ | ☆ | ☆ | － | 8 |
| Lindblad P 1997 | ☆ | － | ☆ | － | ☆ | ☆ | － | ☆ | ☆ | － | 6 |
| Boeing H 1997 | ☆ | － | ☆ | － | ☆ | ☆ | ☆ | ☆ | ☆ | － | 7 |
| Wolk A 1996 | ☆ | ☆ | ☆ | ☆ | ☆ | － | ☆ | ☆ | － | － | 7 |
| Mellemgaard A 1996 | ☆ | － | ☆ | ☆ | ☆ | － | ☆ | ☆ | － | － | 6 |
| Kreiger N 1993 | ☆ | － | ☆ | － | － | ☆ | ☆ | ☆ | ☆ | － | 6 |
| McLaughlin JK 1992 | ☆ | ☆ | ☆ | ☆ | － | ☆ | ☆ | ☆ | ☆ | － | 8 |
| Talamini R 1990 | ☆ | － | ☆ | － | ☆ | ☆ | ☆ | ☆ | ☆ | － | 7 |
| Maclure M 1990 | ☆ | ☆ | ☆ | － | ☆ | ☆ | ☆ | － | ☆ | － | 7 |

**Methodologic quality of cohort studies included in the meta-analysis**

| Study and year | Selection | | | | Comparability | | Outcome | | | Data analysis that used an energy-adjusted residual or nutrient-density model | Total quality scores |
| --- | --- | --- | --- | --- | --- | --- | --- | --- | --- | --- | --- |
|  | Representativeness of the exposed cohort | Selection of the unexposed cohort | Ascertainment of exposure | Demonstration that outcome of interest was not present at start of study | Study controls for age / gender | Study controls for additional factors | Assessment of outcome | Was follow-up long enough for outcomes to occur | Adequacy of follow up of cohorts |  |  |
| Daniel CR 2011 | ☆ | ☆ | ☆ | ☆ | ☆ | ☆ | ☆ | ☆ | ☆ | ☆ | 10 |
| Wilson RT 2009 | ☆ | ☆ | ☆ | ☆ | － | ☆ | ☆ | ☆ | ☆ | － | 8 |
| Wolk A 2006 | ☆ | ☆ | ☆ | ☆ | ☆ | ☆ | ☆ | ☆ | ☆ | ☆ | 10 |
